# MeDiA: Mean Distance Association and Its Applications in Nonlinear Gene Set Analysis

**DOI:** 10.1371/journal.pone.0124620

**Published:** 2015-04-27

**Authors:** Hesen Peng, Junjie Ma, Yun Bai, Jianwei Lu, Tianwei Yu

**Affiliations:** 1 Department of Biostatistics and Bioinformatics, Emory University, Atlanta, Georgia, United States of America; 2 Department of Hematology, Yantai Yuhuangding Hospital, Yantai, Shandong, China; 3 Department of Pharmaceutical Sciences, School of Pharmacy, Philadelphia College of Osteopathic Medicine, Suwanee, Georgia, United States of America; 4 School of Software Engineering, Tongji University, Shanghai, China; 5 Advanced Institute of Translational Medicine, Tongji University, Shanghai, China; University of California, Los Angeles, UNITED STATES

## Abstract

Probabilistic association discovery aims at identifying the association between random vectors, regardless of number of variables involved or linear/nonlinear functional forms. Recently, applications in high-dimensional data have generated rising interest in probabilistic association discovery. We developed a framework based on functions on the observation graph, named MeDiA (Mean Distance Association). We generalize its property to a group of functions on the observation graph. The group of functions encapsulates major existing methods in association discovery, e.g. mutual information and Brownian Covariance, and can be expanded to more complicated forms. We conducted numerical comparison of the statistical power of related methods under multiple scenarios. We further demonstrated the application of MeDiA as a method of gene set analysis that captures a broader range of responses than traditional gene set analysis methods.

## Introduction

In the analysis of high-throughput biological data, such as gene expression data, proteomics data, and metabolomics data, it is often of interest to examine the behavior of groups of variables (genes, proteins or metabolites). The grouping of the variables are commonly pre-determined by functional annotations of the biological units using databases, e.g. Gene Ontology [1] or KEGG pathways [2]. A number of methods were developed in the area of gene set analysis to test for shifts of overall expression levels of genes involved in a gene set under different treatment conditions [3–5]. This approach is commonly referred to as gene set analysis. Besides analyzing the behavior of each gene set in response to certain biological conditions, another class of methods examine the relations between gene sets, both under a single treatment condition [6] and between different treatment conditions [7–9].

So far most of the methods developed for the analysis of gene sets are based on linear relations between random variables. However complex and nonlinear relations between genes and between a gene and treatment condition has been documented [10–12]. Utilizing general probabilistic associations beyond linear association could produce more insights into the data.

If we consider each gene set as a random vector consisting of multiple random variables (genes), seeking association between gene sets boils down to finding probabilistic associations between two random vectors. In this manuscript we first propose and generalize new methods to discover probabilistic association between random vectors. Then we demonstrate the utility of such measures in finding the general dependency between gene sets and multi-dimensional clinical outcomes.

Consider two random vectors ***X*** and ***Y*** and *n* pairs of independent and identically distributed (*i*.*i*.*d*.) random samples ${\left\{x_{i},y_{i}\right\}}_{i=1}^{n}$. We would like to draw inference for the existence of probabilistic association between ***X*** and ***Y*** based on the *n* pairs of samples. The discussion in this paper will focus on the probabilistic association between continuous random variables defined in the Euclidean space.

Classical association statistics like Pearson’s correlation coefficient assume functional forms (for example, piecewise linear, monotonicity) between ***X*** and ***Y***, which are judged as correlated if *Corr*(***X***,***Y***)≠0. Probabilistic association statistic, as the name suggests, perceives associations from the level of probabilistic dependence. That is, ***X*** and ***Y*** are judged as independent if and only if their joint probability density function can be factored, *F*(***X***,***Y***) = *F*_*X*_(***X***)*F*_*Y*_(***Y***), where *F*() is the probability density function for the random vector under consideration. Probabilistic association encapsulates a larger group of associations than traditional correlation coefficient. For example, probabilistic association would consider nonlinear interactions involving multiple variables.

There are multiple methods on probabilistic association discovery linked to functions on the observation distance graph. The distance graph consists of nodes representing each observation *(* ***x***_*i*_, ***y***_*i*_ *)* in the *p*+*q* dimensional Euclidean space. Here *p* and *q* are the dimensions of ***X*** and ***Y***, respectively. Edges of the observation graph would connect two nodes (observations) if specific criteria are satisfied. For example, mutual information and its derivatives have been the most popular probabilistic association statistic to date [11,13]. To estimate mutual information, the joint entropy can be approximated using log-transformed *K*-nearest neighbour distance averaged for each observation [14–16].

Recent breakthrough on distance covariate sheds light on universal association discovery with simplicity and theoretical flexibility [17,18]. Brownian distance covariate was defined as $V_{N}^{2}=\frac{1}{n^{2}}\sum_{k,l=1}^{n}D_{kl}^{X}D_{kl}^{Y}$, where $D_{kl}^{X}$ and $D_{kl}^{Y}$ are linear functions of pairwise distances between sample elements calculated with ***X*** and ***Y*** dimensions, respectively [17]. Given fixed marginal distribution for ***X*** and ***Y***, large Brownian distance covariate suggests the existence of probabilistic association.

In this manuscript, we first describe a general framework for nonlinear association based on the observation graph. It can incorporate different distance metrics and weighting schemes. We then discuss two specific forms of associations including their properties, and illustrate their performance in simulations. Next we show such metrics can be successfully used in nonlinear gene set analysis, which can incorporate multivariate outcome variables, nonlinear associations, and within-gene set heterogeneity.

## Methods

### The general form of association based on observation graph

We propose the general form of association as

$$M=\sum_{k=1}^{n}\sum_{l\neq k}D_{kl}^{\left(X,Y\right)}w_{kl},k=1,\ldots ,n,l=1,\ldots n$$ (1)

where *n* is the total number of data points, $D_{kl}^{\left(X,Y\right)}$ is the distance between data points calculated using both ***X*** and ***Y*** dimensions, and *w*_*kl*_ is the weight depending on the specific considerations of the data. This can be seen as a general framework when we consider different distance metrics and weighting schemes can be used. We describe two specific types of association scores in the following sections.

### Mean Distance Association (MeDiA) score

We let $w_{kl}=\frac{1}{2}{\left(\begin{array}{c} n\\ 2 \end{array}\right)}^{-1}$, then the score is the mean distance between all pairs of points in the joint space of (***X***,***Y***). We are interested in testing the existence of Probabilistic association between two random vectors (***X***,***Y***), given *n* pairs of observations. Consider another pair of random vectors ($\tilde{X}$, $\tilde{Y}$), where $\tilde{X}$ follows independent and identical distribution (*i*.*i*.*d*.) as ***X*** and $\tilde{Y}$ follows *i*.*i*.*d*. distribution as ***Y*.** The only difference is that $\tilde{X}$ and $\tilde{Y}$ are mutually independent. As mentioned above, we would like to compare the sample observation distance from **(*X*,*Y*)** against that from ($\tilde{X}$, $\tilde{Y}$). Intuitively, when ***X*** and ***Y*** are probabilistically associated, the point cloud occupies a smaller space, hence the mean distance tends to be smaller than that from ($\tilde{X}$, $\tilde{Y}$).

#### Theorem 1

*Denote the distance between two independent random samples from (* ***X***, ***Y*** *) as d*_***XY***_, *and the distance between two independent random samples from* ($\tilde{X}$, $\tilde{Y}$) *as* $d_{\tilde{X}\tilde{Y}}$. *Then we have*

$$E\left(d_{XY}\right)\leq E\left(d_{\tilde{X}\tilde{Y}}\right)$$

Proofs of the theoretical results in this section are presented in S1 File. Theorem 1 confirms our intuition: when two random vectors are probabilistically associated, their observations tend to be closer compared with their independent counterparts. Denote distances between two random observations as *d*_*ij*_, where *i* and *j* are the indices among the *n* observations. We have the following property:

#### Corollary 1

For a given observation i, define its mean peer distance as Eq 2. Also define the mean observation distance for n observations as Eq 3:

$${\bar{d}}_{i}=\frac{1}{n-1}\sum_{i\neq j}d_{ij},\;\forall i$$ (2)

$$M=\frac{1}{n}\sum_{i}{\bar{d}}_{i}$$ (3)

Under the null hypothesis that random vectors **X** and **Y** are independent, the mean observation distance follows asymptotic normal distribution as n→∞.

Corollary 1 is easily proved using the Central Limit Theorem. Based on Corollary 1, we can approximate the null distribution of mean distance using normal distribution, which alleviate the computational burden of the permutation test when *n* is reasonably large.

### Mean Distance Association using Nearest Neighbor (MeDiANN)

We let *w*_*kl*_ = 1/*n* when the involved elements are nearest neighbors, and *w*_*kl*_ = 0 otherwise. The association score becomes:

$$\begin{array}{l} {\tilde{d}}_{i}=min\left(d_{i1},\ldots ,d_{in}\right)\\ M=\frac{1}{n}\underset{i}{\sum }{\tilde{d}}_{i} \end{array}$$ (4)

MeDiANN also enjoys asymptotic normality following Bickel and Breiman [19]. That is, regardless of the joint distribution of (***X*, *Y***) or the norm used to define the distance, the MeDiANN score *M* follows

$$\frac{1}{\sqrt{n}}\left(M-E\left(M\right)\right)\to N\left(0,\sigma^{2}\right)\;\;as\;n\to \infty$$

where *E(M)* is the expectation of the MeDiANN score *M*, and σ is the asymptotic standard deviation. This property leads to the proposal of Gaussian plug-in permutation test in the next section, and reduces the computational burden of simulating the score distribution under the null hypothesis of independence between ***X*** and ***Y***.

### Using Gaussian plug-in permutation test for inference

Following theoretical results from the sub-sections above, we propose a permutation test of probabilistic association using MeDiA or MeDiANN. Given *n* pairs of observations ${\left\{x_{i},y_{i}\right\}}_{i=1}^{n}$, the null distribution of the test statistic is generated with the following procedure:

1. Permute relative indices of samples ${\left\{x_{i},y_{i}\right\}}_{i=1}^{n}$, i.e. generate random pairing between the ***x***’s and ***y***’s, and calculate MeDiA or MeDiANN score after permutation.
2. Repeat the above step *K* times and record all the scores, denoted as ${\left\{{\hat{M}}_{k}\right\}}_{k=1,\ldots ,K.}$ Calculate mean and and standard deviation of ${\left\{{\hat{M}}_{k}\right\}}_{k=1,\ldots ,K}$, denoted as $\left(\hat{\mu },\hat{\sigma }\right)$.
3. Approximate the null distribution using normal distribution with mean and standard deviation equaling to $\left(\hat{\mu },\hat{\sigma }\right)$.
4. Compare the score *M* from the actual data with the approximated null distribution, and generate one-sided *p*-value of the test, $p=\Phi \left(\frac{M-\hat{\mu }}{\hat{\sigma }}\right)$.

## Results

### A simple example

**Fig 1:**
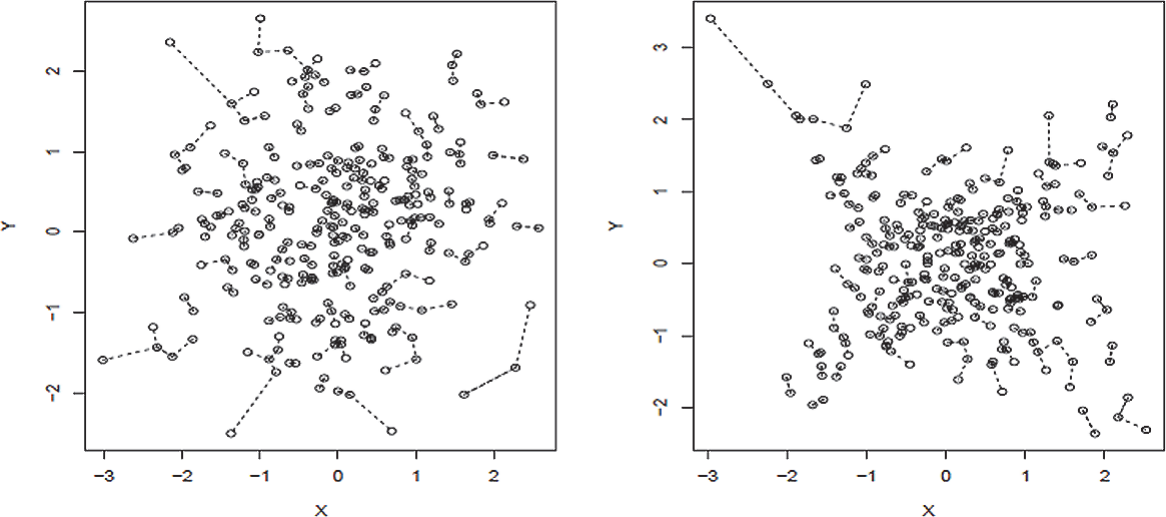
Random samples generated from independent bivariate normal distribution (left), and mixture bivariate normal distribution with ±0.8 covariates (right). The dashed lines connects two observations if they are nearest neighbors.

We show a simple example in Fig 1. Both plots show 300 samples from two bivariate random vectors. Observations on the left panel were sampled from independent bivariate normal distributions. Observations on the right panel were sampled from a mixture bivariate normal distribution. Half of the samples were from a bivariate normal distribution with variance-covariance matrix of $\left(\begin{array}{cc} 1 & 0.8\\ 0.8 & 1 \end{array}\right)$, and the other half of the samples were from a bivariate normal distribution with variance-covariance matrix of $\left(\begin{array}{cc} 1 & -0.8\\ -0.8 & 1 \end{array}\right)$.

Both distributions in Fig 1A and 1B have standard normal marginal distribution and zero correlation coefficient. However, the two samples differ on a group of metrics defined on the observation distances. Table 1 shows the mean distance, mean nearest neighbor distance, and mean log-nearest neighbor distance are all smaller for the dependent case (Fig 1B) compared with independent case (Fig 1A).

**Table 1:** Comparison between the independent bivariate normal distribution and mixture normal distribution in Fig 1.

Metric	Left (Independent)	Right (Mixed Normal)
mean distance (MeDiA)	1.81	1.70
mean nearest neighbor distance (MeDiANN)	0.14	0.12
mean log(*nearest neighbor distance*)	-2.24	-2.43

### Numerical Comparison

For a more systematic assessment of the performance of the proposed method, we conducted a simulation study to examine the size and statistical power of the tests, together with two commonly used non-linear association statistics—the mutual information (MI) and the Brownian covariance (dCov).

The power of the above methods are compared using simulation under scenarios below:

- **Linear association**: *X* and *Y* are both *p*-dimensional random vectors. (*X*,*Y*) follow multivariate normal distribution with zero mean, unit variance and covariance of *ρ* between all pairs of random variables.
- **Variance association**: *X* follows *p*-dimensional normal distribution with zero mean, unit variance, and zero covariance. *Y* is a *p*-dimensional random vector. ***y***_*i*,*j*_ is sampled from *N(0*, *|* ***x***_*i*,*j*_ *|)*, *i = 1*,*…*,*p*, *j = 1*,*…*,*n*.
- **Sine curve association**: *X* is sampled from *p*-dimensional normal distribution with zero mean, unit variance, and zero covariance. Then ***x***_*i*_ is linearly scaled to between 0 and 2π. $y_{i,j}=\sin\left(x_{i,j}+\xi_{i}\right)+\epsilon_{i,j}$, *i = 1*,*…*,*p*, *j = 1*,*…*,*n*, $\xi_{i}\sim \mathrm{Unif}\left(0,2\pi \right),\epsilon_{i,j}\sim N\left(0,\sigma^{2}\right).$ **Square function association** *X* follows *p*-dimensional normal distribution with zero mean, unit variance, and zero covariance. $y_{i,j}=x_{i,j}^{2}+\epsilon_{i,j}$, *i = 1*,*…*,*p*, *j = 1*,*…*,*n*, $\epsilon_{i,j}\sim N\left(0,\sigma^{2}\right).$

For each of the above scenarios, we generated *n* pairs of random samples. We tested the existence of association using MeDiA, MeDiANN, MI and dCov. We used *p = 3* for all cases. The sample size *n* ranged from 25 to 500. For the linear association case, we used *ρ = 0*.*5*, which is a relatively weak pairwise correlation level in real data. For the sine curve and square function associations, a noise term was involved. We used a noise level such that the signal variance is half that of the noise, which is a relatively high noise level in real data. For each scenario/method/sample size tuple, we repeated the simulation 400 times. The *p*-value for each simulation was recorded. And finally the power for each method under each scenario and sample size combination is calculated as the percentage of tests with *p*-values smaller than 0.05.

Power comparison shows differentiated method performance in different scenarios. MeDiANN is defined as average nearest neighbor observation graph edge length. The version of MI used here is estimated as average log-transformed nearest neighbor edge length. They share similar power possibly due to the similar forms of estimation. Nonetheless, there are cases where one is better than the other.

**Fig 2:**
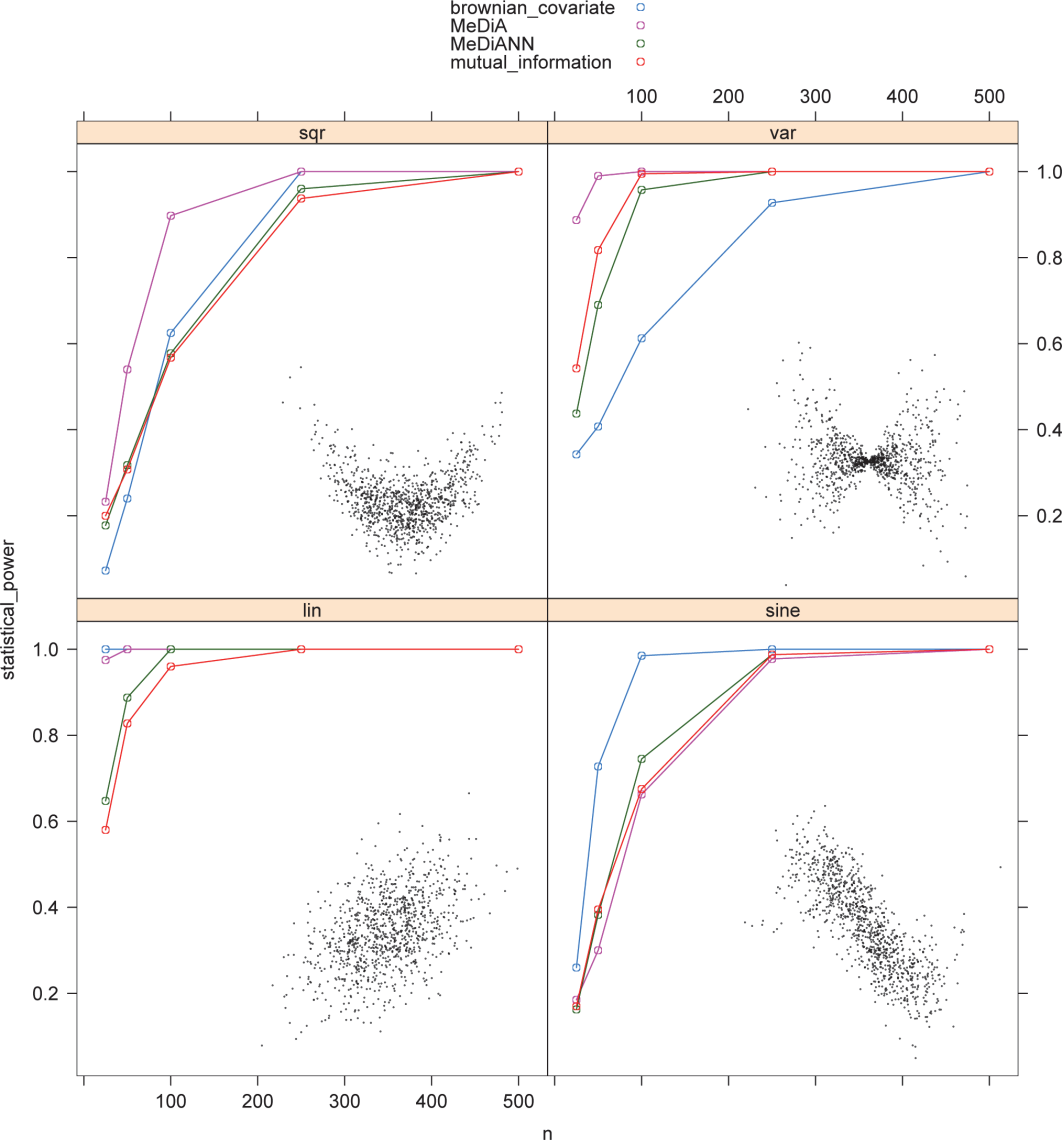
Comparison of statistical power under different scenarios.

In the linear association scenario, dCov and MeDiA performed almost identically. Both are better than the other two methods (Fig 2, lower-left panel). There is a tight connection between Pearson correlation and Brownian Covariate under multivariate normal distributions [17]. At the same time, the data points generated from the joint density function tend to be closer to the diagonal compared to the independent case, causing the average pairwise distance to be smaller, which allows sufficient power for MeDiA. The average distance to the nearest neighbor is reduced not as much as the average pairwise distance. Thus the MiDiANN and MI showed lower power than dCov and MeDiA when the sample size is not large.

In the square function association case, MeDiA outperformed the other three methods (Fig 2, upper-left panel). To understand the result, we consider a simple case where both ***X*** and ***Y*** are one-dimensional. Because the data points fall close to a curve *y = x* ^*2*^, compared to the independent case, a point *(x*_*i*_, *y*_*i*_ *)* is not only closer to other points with similar *x* values, but also to other points with *x* values close to-*x*_*i*_. Thus the average distance utilized by MeDiA is substantially reduced. On the other hand, for dCov, two *y* values that are close may mean the corresponding *x* values are either close or opposite. Thus dCov showed lower power than MeDiA. Similar to the linear case, the reduction of a point’s distance to its nearest neighbor is not very substantial compared to the independent case. Thus the power of MeDiANN and MI trailed the other two methods.

In the variance association case, MeDiA again led the performance, followed by MeDiANN and MI, while dCov trailed in the performance (Fig 2, upper-right panel). As illustrated by the one-dimensional case, the data points tend to stay in a subspace, and the majority of the data points are close to the center. Clearly the average pairwise distance tends to be much smaller than the independent case, favoring MeDiA. At the same time, as the data points are crowded near the origin, the distance of a data point to its nearest neighbor also tends to be small. Thus MeDiANN and MI have reasonably good performance. On the other hand, data points with similar *y* values may have *x* values that spread over a big range, causing difficulty for dCov.

In the sine function association case, dCov achieved higher statistical power than the other three methods (Fig 2, lower-right panel). At the high noise level in our simulations, the bulk of the data follows a relationship close to linear. Thus dCov showed the highest power as it is favored by linear relations. MiDiANN showed better power than MI at smaller sample size.

Overall, general dependency between groups of variables can take many forms. No method is uniformly better than other methods. Each method is favored by certain types of associations, while having difficulty in other forms of associations. It appears that when the bulk of the point cloud follows a relation that is close to linear, dCov has the highest statistical power. When the underlying function is far from linear, MeDiA has the highest statistical power. MeDiANN and MI appear to handle both cases reasonably well, but don’t excel in either. Between the two, MeDiANN has slightly better power than MI when the relation is close to linear, while MI is better when the underlying function is curved.

### Nonlinear gene set analysis using MeDiA

In this section we apply probabilistic association discovery on the analysis of gene sets. Unlike traditional gene set analysis that seeks gene set-level differential expression [3–5], the class of methods we discussed above seeks to find general dependencies between gene sets and the clinical response variable(s). Using this type of methods allows multi-dimensional clinical outcome, heterogeneity in the genes’ behavior within a gene set, as well as non-linear response between genes and the clinical outcome variables. As MeDiA achieved very good overall statistical power in our simulations, in this section, we use MeDiA for the data analysis.

We studied the dataset of gene expression in primary acute lymphoblastic leukemia (ALL) associated with methotrexate (MTX) treatment [20]. The dataset is GSE10255 from the Gene Expression Omnibus [21] (http://www.ncbi.nlm.nih.gov/geo/query/acc.cgi?acc=GSE10255). The major clinical outcome is the change of circulating leukemia cells after initial MTX treatment. We selected the probesets with known ENTREZ Gene IDs. When a gene was represented by more than one probesets, we merged the corresponding probesets by taking the average expression values of the probesets. The data matrix contained 12704 genes and 161 samples.

We used a two-dimensional outcome—the day 0 white blood cell count, and the day 3 white blood cell count, both on log scale. This type of multi-dimensional outcome is not accommodated by traditional gene set analysis methods. For the gene sets, we used a previously described procedure to reduce redundancy [9,22], and selected a group of Gene Ontology Biological Process (BP) terms that are representative—577 biological process terms that contain a total of 10455 genes. We used MeDiA and its accompanying permutation test procedure to test for association between each gene set and the clinical outcome vector. The p-values were adjusted to False Discovery Rate using the Benjemini and Yekutieli procedure [23].

The top gene sets with FDR<0.01 are shown in Table 2. First we notice a number of immune system GO terms in the table (label 1, Table 2). Besides being an anti-cancer agent, MTX is also used to treat autoimmune diseases. It takes action by inhibiting enzymes in methyltransferase and purine metabolism, hence suppressing immune system function [24]. Because this relation is obvious, we shall skip the discussion of details.

**Table 2:** Gene sets associated with the two-dimensional clinical outcome based on MeDiA.

GO term^*^	FDR	Name
^4^ GO:0019827	1.65E-06	stem cell maintenance
^1^ GO:0050852	1.47E-05	T cell receptor signaling pathway
^3^ GO:0006693	0.00042	prostaglandin metabolic process
^5^ GO:0033627	0.00047	cell adhesion mediated by integrin
^1^ GO:0030183	0.00051	B cell differentiation
^1^ GO:0045058	0.00072	T cell selection
^3^ GO:0009225	0.0027	nucleotide-sugar metabolic process
^1^ GO:0045730	0.0027	respiratory burst
GO:0000122	0.0031	negative regulation of transcription from RNA polymerase II promoter
^2^ GO:0007229	0.0031	integrin-mediated signaling pathway
^6^ GO:0051668	0.0031	localization within membrane
^3^ GO:0006633	0.0038	fatty acid biosynthetic process
^6^ GO:0008105	0.0038	asymmetric protein localization
^1^ GO:0019882	0.0038	antigen processing and presentation
^2^ GO:0043123	0.0038	positive regulation of I-kappaB kinase/NF-kappaB cascade
^2^ GO:0043627	0.0038	response to estrogen stimulus
GO:0001837	0.0047	epithelial to mesenchymal transition
^1^ GO:0006959	0.0047	humoral immune response
GO:0044419	0.0047	interspecies interaction between organisms
^2^ GO:0006469	0.0064	negative regulation of protein kinase activity
^2^ GO:0019221	0.0064	cytokine-mediated signaling pathway
^2^ GO:0019722	0.0064	calcium-mediated signaling
^7^ GO:0015012	0.0066	heparan sulfate proteoglycan biosynthetic process
^3^ GO:0042632	0.0079	cholesterol homeostasis
^1^ GO:0050869	0.0079	negative regulation of B cell activation
^5^ GO:0022407	0.0079	regulation of cell-cell adhesion
GO:0046677	0.0087	response to antibiotic
^8^ GO:0006919	0.0094	activation of caspase activity
GO:0006997	0.0099	nucleus organization

^*^ Superscripts by the GO terms are for easy reference from the main text.

Another observation is that a number of signal transduction pathways are among the top GO terms (label 2, Table 2). Some of the pathways were known to be regulated by MTX. The NF-kappaB activation is suppressed by MTX through the inhibition of IkappaB alpha phosphorylation and degradation [25]. The relation between MTX or leukemia to other pathways are not as clearly documented. But there has been some evidences. For example, PELO, which is involved in both cell-matrix adhesion and integrin-mediated signaling pathway, was found to be differentially expressed in AML [26]. It has been shown that MTX affects human bone cell mechanotransduction by interfering with BMP4, which is involved in both integrin-mediated signaling and regulation of protein kinase activity [27]. CAV1, a gene belonging to the cytokine-mediated signaling pathway, was found to be one of the genes characterizing MTX non-responders in patients with rheumatoid arthritis (RA) [28]. At the same time, CAV1 is considered a general tumor suppressor, the lack of expression of which was implicated in the pathogenesis of many cancers, while the over expression of which has also been associated with tumor progression and metastasis in prostate cancers [29]. With regard to the calcium-mediated signaling pathway, it has been shown that the interaction between CXCR4 and SDF-1 is a key mediator of the resistence to chemotherapy in children with ALL [30]. In mouse experiments, MTX treatment caused reduced CXCR4 and CXCL12 expression [31].

Four metabolism GO terms were among the list (label 3, Table 2), three of which were lipid metabolism pathways. It has recently been shown that MTX potentiated glucose uptake and lipid oxidation in skeletal muscle [32]. MTX was also found to improve lipid parameters and fasting plasma glucose levels in a cross-sectional study of humans [33]. The specific pathways detected by our method could indicate certain mechanistic links to explain the observations.

The GO term “stem cell maintenance” is the most significant in the list (label 4, Table 2). Human hematopoietic stem cell maintenance mediated by the transcriptional coactivator CITED2 contributes to leukemia maintenance [34]. In addition, in AML, the loss of Leo1 leads to down-regulation of SOX2 and SOX4, potent oncogenes in myeloid transformation [35]. Two terms involved in cell adhesion were found in the list (label 5, Table 2). There have been known links between leukemia or MTX to cell adhesion. A few genes in the collagen metabolism pathway are altered with leukemia [36], and the overall expression level of collagen increases with MTX treatment [37]. In addition, several cellular adhesion molecules are known to be influenced by MTX [24]. A number of proteins in the membrane organization process are influenced by leukemia (label 6, Table 2) [36,38]. Overall, almost all the GO biological processes found by MeDiA make biological sense, indicating MeDiA is an effective method for nonlinear gene set analysis.

### Differential pathway interaction network discovery

The change of pathway interactions under different cell status is of crucial interest in biomedical study. For example, certain interactions between pathways may be amplified or suppressed in disease state compared with healthy states. These changes in interaction may facilitate the discovery of cell regulatory mechanism. We applied a network reverse engineering procedure for pathway interaction to celiac disease data (NCBI data set GDS3646) and lung cancer data (NCBI data set GDS2771), in which we were specifically interested in identifying pathway interactions that are amplified or suppressed in the disease state.

The celiac disease data consists of gene expression levels of untouched primary leukocytes from 132 unrelated celiac disease individuals and 22185 probesets [39]. Of the 132 individuals, 110 have sustained celiac disease, and 22 are healthy control individuals. Illumina HumanWGv2 annotation data was used to group probe reads into 214 KEGG pathway groups, covering 5201 genes of the data set. The lung cancer data consists of gene expression levels of large airway epithelial cells from cigarette smokers without cancer, with cancer, and with suspect lung cancer [40]. The probe reads were grouped into 214 KEGG pathways using Affymetrix Human Genome U133A database.

The amplified/suppressed pathway interactions were identified using the following procedure:

1. For each pair of pathways *i* and *j* that don’t share any gene, a MeDiANN permutation test of association was applied for the disease group and control group, respectively. Denote the test p-value for disease group as $p_{ij}^{D}$, and $p_{ij}^{C}$ for control group.
2. Rank $\left\{p_{ij}^{D}\right\}$ and $\left\{p_{ij}^{C}\right\}$ from all *i*, *j* combinations in ascending order, and obtain the ranks $\left\{r_{ij}^{D}\right\}$ and $\left\{r_{ij}^{C}\right\}$. This is done for the two groups separately.
3. Calculate between state rank differences $d_{ij}=r_{ij}^{D}-r_{ij}^{C}$.
4. Pathway pairs with rank change *dij* smaller than the 1% quantile in all {*d*_*ij*_} are identified as amplified in association at the disease state. Pairs with *dij* greater than 99% quantile are identified as suppressed in association in disease state.

The identified pathways interactions are then checked for their biological meanings and discussed below.

#### Celiac Disease Pathway Interaction

The newly developed method was used to analyze the publically available Gene Expression Omnibus (GEO) data set GDS3646 (http://www.ncbi.nlm.nih.gov/sites/GDSbrowser?acc=GDS3646). GDS3646 record is an expression analysis of untouched primary leukocytes from unrelated celiac disease individuals [39]. In the study, the gene expression in untouched primary leucocytes from individuals with celiac disease were compared with an EBV-transformed HapMap B cell line data. Celiac disease, a multifactorial disorder with complex genetics, is an enteropathy caused by autoimmune response against wheat gluten, the protein component of the cereals wheat, rye and barley in genetically susceptible individuals [41]. Patients with celiac disease have a wide spectrum of gastrointestinal and extraintestinal manifestations, characterized by intestinal malabsorption and atrophy of intestinal villi [42,43]. Celiac patients experience altered carbohydrate, lipid, peptide/protein, metabolism levels. Untreated celiac patients oxidize more carbohydrates as energy substrate compared to treated subject [42].

**Fig 3:**
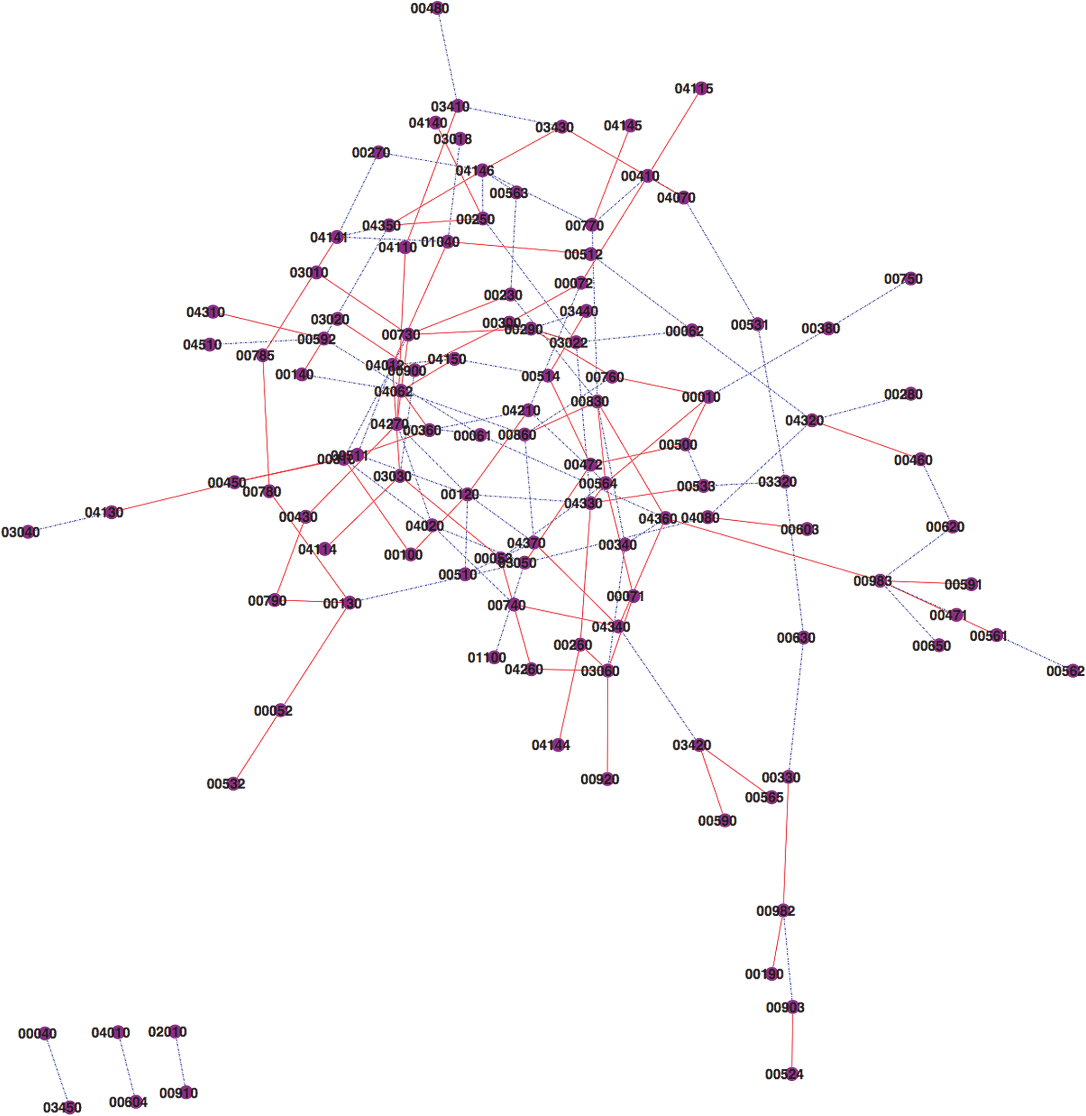
Network interaction for celiac disease pathways. Red edge indicates that the interaction between connected pathways are amplified in disease individuals. Blue edge indicates the interaction suppressed in disease individuals.

The amplified pathway pairs are predominantly related to nutrition absorption and metabolism, while a large proportion of the suppressed pathway pairs are between metabolism and signal transduction (Fig 3; S1 Table). Other pathways potentially linked with celiac disease were also identified. For example, the 04062 chemokine signaling pathway has 6 connections. Chemokines are small peptides that provide directional cues for the cell trafficking and thus are vital for protective inflammatory immune response that requires the recruitment of leukocytes to the site of inflammation upon foreign insult. Celiac disease is known to be an inflammation disease caused by dietary gluten. In genetically predisposed people, gliadin peptides (derivatives of gluten) provokes immune response, which leads to the production of pro-inflammatory cytokines and subsequent damage to, and increased permeability of the intestinal epithelium [44–46].

Another example is the 02010 ABC transporters pathway. The results show that the interaction between ABC transporter pathway and nitrogen metabolism pathway is suppressed in the celiac disease patients. The ATP-binding cassette (ABC) transporters are protein families that couple ATP hydrolysis to activate transport of a wide variety of substrates such as ions, sugars, lipids, sterols, peptides, proteins, and drugs [47,48]. ABC transporters have been confirmed to be related to celiac disease. It has been reported that a close association exists between polymorphism of TAP1 and TAP2 (ABC transporter gene) and disease susceptibility among southern European populations [49]. The products of TAP1 and TAP2 are ABC transporters, which are believed to transport antigenic peptides from the cytoplasm into the endoplasmic reticulum. It was reported that nitrogen balance was modulated in celiac patients [50]. In addition, both nitrate/nitrite are transported by ATP-binding cassette (ABC) transporters [51].

In addition, the relation between 04370 VEGF signaling pathway and several pathways is found to be modulated in celiac patients, including 04340 hedgehog signaling, 510 N-Glycan biosynthesis, 00860 porphyrin and chlorophyll metabolism, 00120 primary bile acid biosynthesis. Vascular endothelial growth factor (VEGF) family and its receptor systems have been demonstrated to be the fundamental regulator in the cell signaling of angiogenesis. Angiogenesis is an essential biological process involved in the progression of a variety of major diseases such as cancer, diabetes and inflammation [52]. It was reported that small-bowel mucosal vascular network was altered in untreated coeliac disease. The study found that on a gluten-containing diet the mucosal vasculature in the small intestine of untreated coeliac disease patients was altered in overall organization as well as in the number and maturity of the vessels when compared to healthy subjects. In patients on a gluten-free diet, the vasculature normalized parallel to mucosal recovery [53]. Angiogenesis is reported to be related to hedgehog signaling [54,55], bile acid[56], glycan biosynthesis[57], porphyrin [58,59]. 04210 apoptosis pathway, the programmed cell death, also shows a number of connections. Much evidence supported the increase of small intestinal apoptosis in celiac disease [60]. Some other study demonstrated that enterocyte apoptosis induced by activated intraepithelial lymphocytes is increased in celiac disease [61].

#### Lung Cancer Pathway Interaction

The newly developed method is also tested on GDS2771 data set (http://www.ncbi.nlm.nih.gov/sites/GDSbrowser?acc=GDS2771), which is the microarray data of large airway epithelial cells from cigarette smokers without cancer, with cancer, and with suspect lung cancer. Many studies demonstrated the correlation of altered metabolism with lung cancer, including basal metabolism [62–64], carbohydrate metabolism[65,66], protein metabolism[66–68], lipid metabolism[69], and xenobiotic metabolism[70].

**Fig 4:**
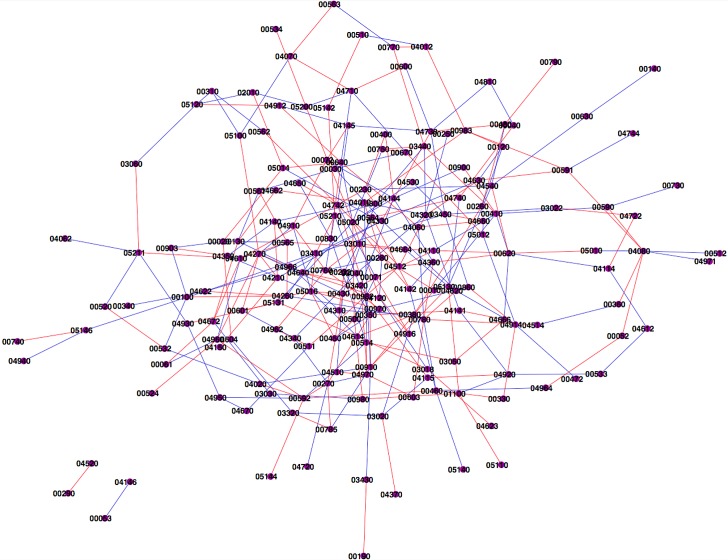
Network interaction for lung cancer pathways. Red edge indicates that the interaction between connected pathways are amplified in disease individuals. Blue edge indicates the interaction suppressed in disease individuals.

The result show that many relations between metabolism related pathways are regulated (Fig 4; S2 Table). Take the TCA pathway as an example, the citrate cycle (TCA cycle, Krebs cycle) is an important aerobic pathway for the final steps of the oxidation of carbohydrates and fatty acids. Modulation of TCA cycle enzymes have been demonstrated in lung cancer. Decreased activities of TCA cycle key enzymes were observed in lung cancer bearing animals [71].

Some specific pathways related to lung cancer are also caught on the list: It was shown that the correlation between pathway 00072, the synthesis and degradation of ketone bodies pathway and 04145, the phagosome pathway is amplified in lung cancer patients. Phagocytosis is the cellular process of engulfing solid particles by the cell membrane to form an internal phagosome, which is a central mechanism in both immune and apoptosis responses. There is a broadly accepted view that bronchial neoplasms or its products suppress phagocytic functions of alveolar macrophages [72]. The alveolar macrophage is believed to be of central importance in the immune response against infection and tumor. It has been reported that there are type-specific alterations in phagocytosis ability of alveolar macrophage in lung cancer patients, which may result in an inability to stimulate anti-tumor immunity and subsequently cause observed differences between lung cancer subgroups. Altered blood monocyte (BM) phagocytosis ability was also observed in patients with lung cancer [73,74]. More importantly, some studies proved that ketone bodies affect the phagocytic activity of macrophages and leukocytes [75].

Another interesting example is that the correlation between 00232, the pathway of caffeine metabolism, and 00760, the nicotinate and nicotinamide metabolism, amplified in smokers with lung cancer. First of all, both caffeine and nicotine metabolism are generally believed to related to the risk of lung cancer. Cigarette smoking is a clear risk factor for lung cancer. Even though nicotine, one of the major ingredients and the causative agent for addiction of cigarette smoking, is generally believed not a carcinogen by itself. However, several studies have shown that nicotine can induce cell proliferation and angiogenesis [76]. Nicotine metabolism by cytochrome P450 2A6 (CYP2A6) varies across ethnicity and race, which is indicated to be related to smoking behavior and lung cancer risk [77,78]. The same as smoking, the consumption of coffee is a very old and popular habit. Coffee contains catechins and flavonoids, which exhibit anti-carcinogenic properties. Conversely, caffeine may elevate cancer risk through a variety of mechanisms [79,80]. Caffeine, an environmentally prominent phosphodiesterase, has been proved to selectively stimulate the growth of pulmonary adenocarcinoma and small airway epithelial cells [81]. Not only are both nicotine and caffeine related to lung cancer, but also many evidences suggested that the metabolism of caffeine and nicotine are closely correlated. Caffeine is mainly metabolized by cytochrome P450 1A2 (CYP1A2). Actually caffeine metabolism has been used as an in vivo marker of CYP1A2 activity, which has been clearly demonstrated to be induced by cigarette smoking [82]. The difference of caffeine intake and plasma concentrations among smokers and nonsmokers was reported [83]. The results from 69 US samples showed that smokers had significantly higher caffeine intake than nonsmokers and the ratio of concentration/dose of caffeine was approximately four-fold higher in nonsmokers than in smokers [83]. In animal studies, nicotine have been proved to induce the activity of several enzymes, including CYP1A2 [84]. It explains why nonsmokers have high plasma caffeine concentration after intake of the same dose of caffeine compared to smokers. Some other research articles reported that the combined NAT2/CYP1A2 status was related to lung adenocarcinoma [78].

## Discussion

In this paper we have discussed the general theory and applications of association discovery using functions on the observation graph. Statistics of similar form to Eq 1 are capable of detecting associations between continuous random vectors using permutation test of association (Table 3). However, we would like to point out that Eq 1 is only one of the ways to test probabilistic association. For example, Brownian distance covariate (dCov) utilizes the covariates of between-observation distances calculated using either random vector. Its estimation is derived from estimating the L2 distance between characteristics functions of joint distribution and product of marginal distributions. It arrives at an observation distance product form. This is different from Eq 1. We are confident that there are way more methods to test probabilistic associations to be discovered.

**Table 3:** Summary of methods on Probabilistic association discovery discussed in this paper.

**Name**	**Statistic**	**Inference**
MeDiA	Mean distance	Permutation Test
MeDiANN	Mean nearest neighbor distance	Permutation Test
Mutual Information	Mean log nearest neighbor distance	Permutation Test
Brownian Cov	Distance covariate	Permutation Test

Mutual information estimation is derived from the estimation of joined entropy of variables under consideration. There are two estimation methods generally used. (1) Reverse engineering of joint density. This is the most popular method but is generally not applicable when the dimension under consideration goes too high. (2) Estimation of joint entropy using graph distance. This method as mentioned in the article can circumvent joint density estimation and is more appropriate in high dimensional data analysis.

We have generalized the estimation of mean observation distance (MeDiA), mean nearest neighbor observation distance (MeDiANN), and mutual information (MI) estimate into the same framework of functions on the observation graph. Simulation study showed that the three statistics have different performance in terms of statistical power under different scenarios. In hindsight, we realized that: testing of probabilistic association using observation distance under the framework of Eq 1 actually rests on the testing of observation distance distributions. More specifically, when random vectors ***X*** and ***Y*** under consideration are associated, their distribution of observation distance should be different from their independent counterpart $\tilde{X}$ and $\tilde{Y}$. In our upcoming work, we will explore an omnibus probabilistic association test based on the observation distance distribution. We would expect this test to be more flexible compared with the methods compared in this paper.

On the applications front, probabilistic dependency discovery methods such as MeDiA and Brownian Covariate can be used to test the hypothesis that a gene set is associated with clinical outcome variables. The association can involve multiple clinical variables, nonlinear interaction, and be heterogeneous within the gene sets. We have also successfully applied the MeDiA for the detection of differential interaction between gene sets under different treatment conditions.

## Conclusions

Overall, with the complexity of the biological system, and the documented presence of nonlinear and conditional dependencies, MeDiA and other probabilistic dependency discovery methods based on the observation graph are useful in unraveling high-throughput data to make new biological discoveries.

## Supporting Information

S1 File Mathematical proof of Theorem 1. (PDF)

S1 Table Pathway pairs with amplified or suppressed relations in dataset GDS3646. (XLSX)

S2 Table Pathway pairs with amplified or suppressed relations in dataset GDS2771. (XLSX)
