# Supplementary material for: MeDiA: Mean Distance Association and Its Applications in Nonlinear Gene Set Analysis
Source: PLoS One. 2015 Apr 27;10(4):e0124620. doi: 10.1371/journal.pone.0124620 (PMC4411044; doi:10.1371/journal.pone.0124620)
Supplement: S1 File — (PDF) [file pone.0124620.s001.pdf]

1 Proof of Theorems

Theorem 1. We would like to make the following definitions before proceeding:

- (X', Y'): follows *i.i.d.* as (X, Y)
- $(\hat{X}, \hat{Y})$: $\hat{X}$ follows *i.i.d.* as X; $\hat{Y}$ follows *i.i.d.* as Y. But $\hat{X}$ and $\hat{Y}$ are independent. $(\hat{X}, \hat{Y})$ is independent of all previously defined random variables.
- $(\hat{X}', \hat{Y}')$: follows *i.i.d.* as $(\hat{X}, \hat{Y})$ and is independent of all the above random variables.

Based on the abvoe definition, the distance between independent random pairs of observations from (X, Y) and its independent counterpart $(\hat{X}, \hat{Y})$ can be expressed as:

$$\begin{aligned} E(d_{XY}) &= E(|(X,Y) - (X',Y')|_{p+q}) \\ E(d_{\hat{X}\hat{Y}}) &= E(|(\hat{X},\hat{Y}) - (\hat{X}',\hat{Y}')|_{p+q}) \end{aligned}$$

Further more, based on Equation 2.5 of [1], we have:

$$\begin{aligned} E(|(X,Y) - (X',Y')|_{p+q}) &= \int_{R^{p+q}} \frac{1 - |f_{X,Y}(s,t)|^2}{C_{p+q}|(t,s)|_{p+q}^{1+p+q}} d(s,t) \\ E(|(\hat{X},\hat{Y}) - (\hat{X}',\hat{Y}')|_{p+q}) &= \int_{R^{p+q}} \frac{1 - |f_{\hat{X},\hat{Y}}(s,t)|^2}{C_{p+q}|(t,s)|_{p+q}^{1+p+q}} d(s,t) \end{aligned}$$

where $f_{X,Y}(s,t)$ is the characteristics functions of (X, Y); $f_{\hat{X},\hat{Y}}(s,t)$ is the characteristics functions of $(\hat{X}, \hat{Y})$; C_{p+q} is a constant defined in Equation 2.3 of [1].

Meanwhile, the characteristics function of $(\hat{X}, \hat{Y})$ can be factored because of their independence

$$f_{\hat{X},\hat{Y}}(s,t) = f_{\hat{X}}(s) f_{\hat{Y}}(t)$$

Summarizing the above, we have

$$\begin{aligned} E(d_{XY} - d_{\hat{X}\hat{Y}}) &= \int_{R^{p+q}} \frac{|f_X(s)|^2 |f_Y(t)|^2 - |f_{XY}(s,t)|^2}{C_{p+q}|(t,s)|_{p+q}^{1+p+q}} d(t,s) \\ &\leq 0 \end{aligned}$$

according to Cauchy-Schwarz inequality. □

References

[1] G. J. Szkely, M. L. Rizzo, and N. K. Bakirov. Measuring and testing dependence by correlation of distances. *The Annals of Statistics*, 35(6):2769–2794, 12 2007.
